# Comparative analysis of stripe rust resistance in seedling stage and *Yr* gene incidence in spring and winter wheat from Xinjiang, China

**DOI:** 10.3389/fpls.2024.1394213

**Published:** 2024-05-01

**Authors:** Hanlin Lai, Yuyang Shen, Hong Yang, Dilantha W. G. Fernando, Chenrong Ren, Feifei Deng, Yi Lu, Na Sun, Li Chen, Guangkuo Li, Huiqing Wang, Haifeng Gao, Yue Li

**Affiliations:** ^1^ College of Life Science, Xinjiang Agricultural University, Urumqi, China; ^2^ Institute of Plant Protection, Xinjiang Academy of Agricultural Sciences/Key Laboratory of Integrated Pest Management on Crop in Northwestern Oasis, Ministry of Agriculture and Rural Affairs, Urumqi, Xinjiang, China; ^3^ Department of Plant Sciences, University of Manitoba, Winnipeg, MB, Canada; ^4^ Plant Protection Station of Xinjiang Uygur Autonomous Region, Urumqi, Xinjiang, China; ^5^ Institute of Agricultural Sciences of Ili Prefecture, Ili, Xinjiang, China

**Keywords:** wheat variety, disease resistance, molecular detection, *Puccinia striiformis* f. sp. *tritici*, Xinjiang

## Abstract

**Background:**

Stripe rust, caused by the fungus *Puccinia striiformis* f.sp. *tritici* (*Pst*), poses a significant threat to global wheat production.

**Objectives:**

This study aims to analyze the distribution of stripe rust resistance genes, characterize resistance phenotypes at the seedling stage of 137 spring and 149 winter wheat varieties in Xinjiang, China, and discern differences in resistance between spring and winter wheat varieties.

**Design:**

We used various Pst races (CYR23, CYR29, CYR31, CYR32, CYR33, CYR34) to characterize seedling resistance of spring and winter wheat varieties and to correlate resistance to the presence of wheat resistance genes (*Yr5*, *Yr9*, *Yr10*, *Yr15*, *Yr17*, *Yr18*, *Yr26*, *Yr41*, *Yr80*, *Yr81*) using molecular markers.

**Results:**

Among spring wheat varieties, 62, 60, 42, 26, 51, and 24 varieties exhibited resistance to CYR23, CYR29, CYR31, CYR32, CYR33, and CYR34, respectively, with four varieties resistant to all varieties. Among winter wheat varieties, 66, 32, 69, 26, 83, 40 varieties demonstrated resistance to CYR23, CYR29, CYR31, CYR32, CYR33, and CYR34, respectively, with four varieties resistant to all varieties. Molecular testing revealed that, in spring wheat, 2, 17, 21, 61, 10, 0, 10, 79, and 32 varieties carried *Yr9*, *Yr10*, *Yr15*, *Yr17*, *Yr18*, *Yr26*, *Yr41*, *Yr80*, and *Yr81* genes, respectively. In winter wheat, 40, 20, 7, 143, 15, 1, 6, 38, and 54 varieties carried *Yr9*, *Yr10*, *Yr15*, *Yr17*, *Yr18*, *Yr26*, *Yr41*, *Yr80*, and *Yr81* genes, respectively. Notably, winter wheat exhibited a significantly higher resistance frequency than spring wheat, particularly in the incidence of *Yr9*, *Yr10*, *Yr17*, *Yr18*, and multi-gene combinations.

**Conclusion:**

In summary, this study provides information on seedling stage resistance to stripe rust 286 Xinjiang wheat varieties, elucidates the distribution of resistance genes in this population, and offers a mechanistic basis for breeding durable resistance in wheat. varieties from Xinjiang.

## 1 Introduction

China is the world’s largest producer of wheat, generating 128 million metric tons per annum (Zhao and Kang, 2023). In China, wheat is cultivated across multiple environmentally unique and geographically isolated regions, giving rise to multiple strains of the parasitic fungus, *Puccinia striiformis* f. sp. *tritici* (*Pst*), the causal agent of wheat stripe rust (Zeng and Luo, 2006; Wan et al., 2007; Zhan et al., 2016; Ma et al., 2023). *Pst* is an ancient airborne pathogen that specifically colonizes wheat during its asexual life cycle (Brown and Hovmoller, 2002; Schwessinger, 2017; Li et al, 2021; Zhao et al., 2013), and *Pst* urediniospores are dispersed via wind, resulting in specific races localizing to certain regions (Yao et al., 2019a). Virulent strains of *Pst* cause economically devasting stripe rust outbreaks, which resulted in an average yield loss of 1.54 million metric tons per year from 2000 to 2018 in China alone (Zhang et al., 2022). Wheat stripe rust has been responsible for several pandemics in China, most recently in 2017, which resulted in massive yield losses and significant economic impacts (Shen et al., 2002; Wan et al., 2007; Chen et al., 2009; Ma, 2018).

Local varieties of wheat are often referred to as landrace varieties. These are crop composite populations that have been adapted to specific climatic and geographic conditions of a particular region over a long period of cultivation, and they are relatively genetically stable (Shen et al., 2002). Xinjiang Province is found far northwest of China, bordering Tibet, and is classified as an independent epidemic area of wheat stripe rust (Zeng and Luo, 2006). In China, wheat stripe rust has the characteristics of high epidemic frequency, wide occurrence range, and severe damage to wheat production (Jiang et al., 2022). Xinjiang wheat appears to have originated through natural hybridization between Polish and common wheat, suggesting that local wheat was likely introduced and selectively bred within the region itself rather than through the domestication of wild wheat (Betts et al., 2014). Furthermore, Xinjiang’s distinctive climate allows for the cultivation of both oversummer and overwinter wheat, providing year-round hosts for *Pst*. Xinjiang is also home to Chinese *Berberis*, or the *barberry* plant, the host required for the sexual reproduction phase of the *Pst* lifecycle, indicating that Xinjiang supports significant diversity in *Pst* races (Zhuang, 2019). To protect wheat crops within Xinjiang, it is critical to determine the resistance distribution of local wheat cultivars to prevalent races of *Pst.* Additionally, as wheat varieties expressing a single resistance gene are cultivated over time, selective pressure has driven pathogen evolution to escape gene-for-gene resistance, leading to obsolete resistance genes (Kang et al., 2015; Li et al., 2020). Therefore, Xinjiang’s unique local landraces represent valuable genetic sources for potential resistance genes against *Pst* that can be bred into major cultivars (Mujeeb-Kazi et al., 2013; Chen et al., 2016, Chen et al., 2018; Yao et al., 2019b; Dai et al., 2020).

Despite the effective use of fungicides to combat recent epidemics, deployment of R-genes against virulent *Pst* races remains the most economically and environmentally viable approach (Zhao and Kang, 2023). Currently, the dominant *Pst* races in China, designated with the CYR (Chinese yellow rust) prefix, are CYR32, CYR33, and CYR34 (Zhao and Kang, 2023). Over the last century, the *Yr* wheat resistance genes *Yr9*, *Yr10*, *Yr17*, and *Yr26* have been extensively used in breeding for wheat stripe rust resistance (Han et al., 2010). With the emergence of new virulent races, such as CYR29, CYR31, CYR32, and CYR34, resistance conveyed by *Yr9*, *Yr10*, *Yr17*, and *Yr26* has been revealed (Han et al., 2010). Some all-stage resistance (ASR) genes, such as *Yr5* and *Yr15*, are still effective against prevalent races of *Pst*, but are rarely used in breeding (Zeng et al., 2014). The *Yr18* gene confers non-race specific resistance to stripe rust and slows infection time and spore production, resulting in adult plant resistance (APR) (Singh et al., 2000). Presently, the main race types prevalent in Ili prefecture, the largest wheat-producing region of Xinjiang, are CYR34 and Su 11-1 (Chen et al., 2023). It has been shown that *Yr5* and *Yr15* are highly resistant to stripe rust (Zhou et al., 2023), making the detection of these resistance genes essential for the prevention and control of wheat stripe rust in Xinjiang.

With the expansion of molecular marker technologies, an increasing number of molecular markers are being used in the breeding of disease-resistant wheat varieties. Currently, the major molecular markers based on genomic DNA molecular polymorphisms include sequence tagged sites (STS), simple sequence repeats (SSR), and kompetitive allele-specific polymerase chain reactions (KASP) (Song et al., 2023). By detecting linked molecular markers, disease resistance genes in wheat can be efficiently detected, either directly or indirectly. Identification of cultivars containing effective resistance genes permits selective breeding for robust resistance and enables the stacking of multiple genes to establish long-lasting disease-resistant varieties (Luo et al., 2023). Presently, 83 stripe rust resistance genes (*Yr1*-*Yr83*) have been discovered and conclusively named, and more than 300 genes or QTL have been identified and temporarily named (McIntosh et al., 2009; Li et al., 2020).

A previous study detected a high frequency of *Yr9* in wheat cultivars of the Huang-Huai region, along with significant expression of *Yr18* in Huang-Huai landraces (Huang et al., 2020). Alternatively, the main wheat varieties in southwest China exhibit a high frequency of polygene polymerization (Xi et al., 2021). Landraces in northwest China display high frequency of *Yr9*, which has been widely used for breeding, as well as varieties with multiple *Yr* aggregates (Wang et al., 2018). The ASR genes *Yr5* and *Yr15* are generally absent from wheat panels in China, where only a few reports identified *Yr15* (Li et al., 2016). Regions adjacent to Xinjiang, such as Pakistan and Kazakhstan, have the highest distribution frequency of *Yr18* and *Yr10*, which convey resistance to most races of stripe rust (Sobia et al., 2010; Kokhmetova et al., 2021). The frequencies of *Yr26* and *Yr10* in Indian wheat are 69.2% and 50%, respectively, with some highly resistant varieties containing stacks of 15 *Yr* genes (Rani et al., 2019).

Here, we perform a systematic study of phenotypic variation and molecular characterization of stripe rust resistance of winter and spring wheat verities in Xinjiang. We employ a panel of 149 winter wheat varieties and 137 spring wheat varieties in Xinjiang for the characterization of stripe rust resistance against six races at the seedling stage. We further correlate resistance phenotypes to the prevalence of 10 stripe rust resistance genes, as identified with appropriate molecular markers. While the distribution of wheat stripe rust resistance genes in Xinjiang has been recently evaluated, this study is the first to directly assess resistance phenotypes in tandem with the identification of resistance genes (Zhang et al., 2023). This work provides valuable resources for the identification of breeding targets for enhancing wheat stripe rust resistance from the pool of Xinjiang wheat.

## 2 Materials and methods

### 2.1 Materials

A total of 286 wheat varieties, including 137 spring and 149 winter varieties, were tested. The Spring Wheat Breeding Team of the Grain Crops Research Institute of the Xinjiang Academy of Agricultural Sciences provided the 137 spring wheat varieties. The Wheat Breeding Team of the Agricultural Research Institute of the Ili Region contributed 55 YINONG winter wheat varieties. The Winter Wheat Breeding Team of the Grain Crops Research Institute of the Xinjiang Academy of Agricultural Sciences provided 94 wheat varieties of the winter wheat varieties. Associate Professor Zhan Gangming from the College of Plant Protection, North Agriculture and Forestry University of Science and Technology, provided the control materials, including Mingxian 169 and single gene line materials.

### 2.2 Identification of seedling disease resistance

Phenotyping of seedling diseases was carried out in climatic chambers at the Institute of Plant Protection, Xinjiang Academy of Agricultural Sciences, Xinjiang, China. In brief, 10–15 seeds from each wheat variety were sown in small pots and cultivated indoors until the one-leaf-one-heart stage. Seedlings were inoculated with *Pst* races combined with e-fluoridized solution using a pipetting gun set to 5 μL and then maintained in dark conditions for 24 h (10°C). Afterward, seedlings were transferred to climatic chambers (12-hr light/12-hr dark). When symptoms were fully visible in the control group, Mingxian 169, the infection type (IT) was assessed and classed as high resistance (IT: 0–3), moderate resistance (IT: 4–5), or high susceptibility (IT: 6–9).

### 2.3 Molecular detection of stripe rust resistance genes

Appropriate amounts of wheat leaf tissue were collected, and a modified CTAB method was used for the extraction of genomic DNA (Allen et al., 2006). The quality and quantity of the extracted genomic DNA was determined using a NanoDrop 610 spectrophotometer (ThermoScientific, Wilmington, DE, USA), and the DNA was diluted to 100 ng/μL and stored at -20°C. Polymerase chain reaction (PCR) amplification using specific primers for SSR, STS, and SNP molecular markers associated with *Yr5*, *Yr9*, *Yr10*, *Yr15*, *Yr17*, *Yr18*, *Yr26*, *Yr41*, *Yr80*, and *Yr81* was performed, followed by agarose gel electrophoresis on 1–2% agarose gel. PCR reactions consisted of 2× Easy *Taq* PCR mix (12.5 μL), forward primer (F: 1 μL), and reverse primer (R: 1 μL). PCR reactions involved a 4-minute predenaturation at 94°C, followed by 35 cycles of denaturation at 94°C for 30 seconds, annealing at 55–65°C for 30 seconds, and extension at 72°C for 30–60 seconds. KASP-SNP molecular marker PCR reactions consisted of 2× KASP mix (170 μL), forward primer 1 (F1: 0.51 μL), forward primer 2 (F2: 0.51 μL), and reverse primer (R: 1.36 μL). Subsequently, 1 μL of DNA template was added to each reaction. Primers were synthesized by Shanghai Bioengineering. Refer to **Table 1** for primer sequences.

**Table 1:** Primers used in this study.

Gene	Marker Type	Primer Name	Primer Sequence (5’–3’)	Annealing temperature (°C)	Reference
*Yr5*	SSR	Wmc175	GCTCAGTCAAACCGCTACTTCT	57	(Chen et al., 2003)
			CACTACTCCAATCTATCGCCGT		
	KASP	Yr5F	GAAGGTGACCAAGTTCATGCTGCGCCCCTTTTCGAAAAAATA	touchdown PCR	(Marchal et al., 2018)
		Yr5H	GAAGGTCGGAGTCAACGGATTCTAGCATCAAACAAGCTAAATA		
		Yr5R	ATGTCGAAATATTGCATAACATGG		
*Yr9*	STS	H2O	GTTGTAAGGGAGCTCGAGCTG	57	(Liu et al., 2008)
			GTTGGGCAGAAAGGTCGACATC		
	RAPD	AF1/AF4	GGAGACATCATGAAACATTTG	58	(Francis et al., 1995)
			CTGTTGTTGGGCAGAAAG		
*Yr10*	AFLP	Sc200	CTGCAGAGTGACATCATACA	60	(Shao et al., 2001)
			TCGAACTAGTAGATGCTGGC		
	SSR	Xpsp3000	GCAGACCTGTGTCATTGGTC	57	(Wang et al., 2002)
			GATATAGTGGCAGCAGCAGGATAC		
*Yr15*	SSR	Barc8	GCGGGAATCATGCATAGGAAAACAGAA	57	(Peng et al., 2000)
			GCGGGGGCGAAACATACACATAAAAACA		
	SSR	Xgwm413	TGCTTGTCTAGATTGCTTGGG	60	
			GATCGTCTCGTCCTTGGCA		
*Yr17*	SCAR	VENTRIUP-LN2	AGGGGCTACTGACCAAGGCT	58	(Jia et al., 2010)
			TGCAGCTACAGCAGTATGTACACAAAA		
*Yr18*	Cssfr1	L34DINT9F	TTGATGAAACCAGTTTTTTTTCTA	57	(Lagudah et al., 2009)
		L34PLUSR	GCCATTTAACATAATCATGATGGA		
		L34SPF	GGGAGCATTATTTTTTTCCATCATG	57	
		L34DINT13R2	ACTTTCCTGAAAATAATACAAGCA		
	KASP	Lr34-KASP-E11	GAAGGTGACCAAGTTCATGCTGGGAGCATTATTTTTTTCCATCA	touchdown PCR	(Fang et al., 2020)
			GAAGGTCGGAGTCAACGGATTGGGAGCATTATTTTTTTCCATCT		
			AGCGAATCCAGTATGGAAAT		
*Yr26*	STS	Xwe173	GGGACAAGGGGAGTTGAAGC	61	(Wang et al., 2008)
			GAGAGTTCCAAGCAGAACAC		
*Yr41*	STS	BE446068F	ATGGCTTGGTTTCCCTTTTT	59	(Zhang, 2016)
			TATCAAGCTCGCTCGGCTAA		
*Yr80*	KASP	KASP_53113	GAAGGTGACCAAGTTCATGCTTGTACAATGACTCCTCGACTAACA	touchdown PCR	(Nsabiyera et al., 2018)
			GAAGGTCGGAGTCAACGGATTTGTACAATGACTCCTCGACTAACG		
			GCCACGCAATATCACCATCG		
*Yr81*	KSAP	KASP_3077	GAAGGTGACCAAGTTCATGCTATTCCAAAGTAATTGGCAACAGGTTCA	touchdown PCR	(Gessese et al., 2019)
			GAAGGTCGGAGTCAACGGATTCCAAAGTAATTGGCAACAGGTTCG		
			TGTGGAGCGTGACAATGAGGAAGTT		

### 2.4 Data analysis

UpSetR (version 1.4.0) package in the R environment was used for gene combination analysis, and ggplot2 (version 3.4.4) and venn (version 1.11) packages were used for data processing and graph generation, respectively.

## 3 Results

### 3.1 Resistance of spring and winter wheat seedlings to different strip rust races

**Figure 1:**
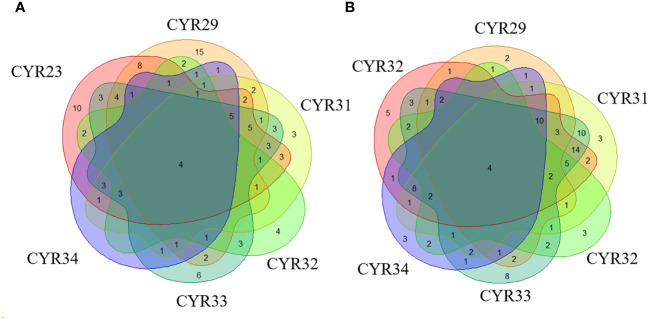
Identification of resistance against six *Pst* races in spring and winter wheat landraces from Xinjiang. **(A)** Depicts the number of varieties resistant to different races in spring wheat, and **(B)** Depicts the number of varieties resistant to different races in winter wheat.

The number of varieties of spring wheat resistant to CYR23, CYR29, CYR31, CYR32, CYR33, and CYR34 amounted to 62 (45.26%), 60 (43.8%), 44 (32.12%), 25 (18.25%), 50 (36.60%), and 25 (18.25%), respectively. Notably, Xinchun No. 32, Xinchun No. 51, Liangchun1723, and Liangchun1817 were resistant to all 6 races, accounting for 2.92% of all resistant varieties (**Figure 1A**; **Supplementary Table S1**).

We identified 62 (44.3%), 32 (21.48%), 70 (46.89%), 26 (17.45%), 84 (56.38%), and 40 (26.85%) winter wheat varieties resistant to CYR23, CYR29, CYR31, CYR32, CYR33, and CYR34, respectively. Among these, varieties 2014-132-4-5, 2014-129-13-9, 6239, and 6444 were resistant to all 6 races, accounting for 2.68% of all resistant varieties (**Figure 1B**; **Supplementary Table S2**).

Presently, the predominant races in Xinjiang are CYR32, CYR33, and CYR34. A detailed analysis of these races has revealed that 11 spring wheat varieties exhibited resistance to CYR33 and CYR34, including Xinchun No. 12, Xinchun No. 14, and Xinchun No. 16. Four varieties, including Xinchun No. 34, Xinchun No. 39, and Liangchun1934, exhibited resistance to CYR33 and CYR32. Another four varieties, including 2016, Liangchun1758, and Liangchun1832, demonstrated resistance to CYR32 and CYR34. Eight varieties, including Xinchun No. 29, Heli1881, and Liangchun547, exhibited resistance to all three races.

Among the winter wheat varieties, 24 varieties, including Zhaonong147, 2014-132-10-10, and 2014-12-3-1, demonstrated resistance to both CYR33 and CYR34. Twelve varieties exhibited resistance to CYR33 and CYR32, including 2014-129-1-6, Pin I-1, and Pin I-8. Additionally, 2 varieties, namely Jindong008 and Tiandong33, exhibited resistance to both CYR32 and CYR34, whereas 7 varieties, including 2014-132-10-6, 6222, and 6238, demonstrated resistance to all three races. Overall, Xinjiang wheat exhibits the highest frequency of resistance to CYR33 among the Su 11 taxa. Previous surveys have also revealed the highest proportion of Su 11 pathogenic taxa of stripe rust (Chen et al., 2023; Ma et al., 2023). Consequently, Xinjiang wheat possesses resistance to the currently prevalent races.

### 3.2 Comparison of disease resistance between spring and winter wheat

**Figure 2:**
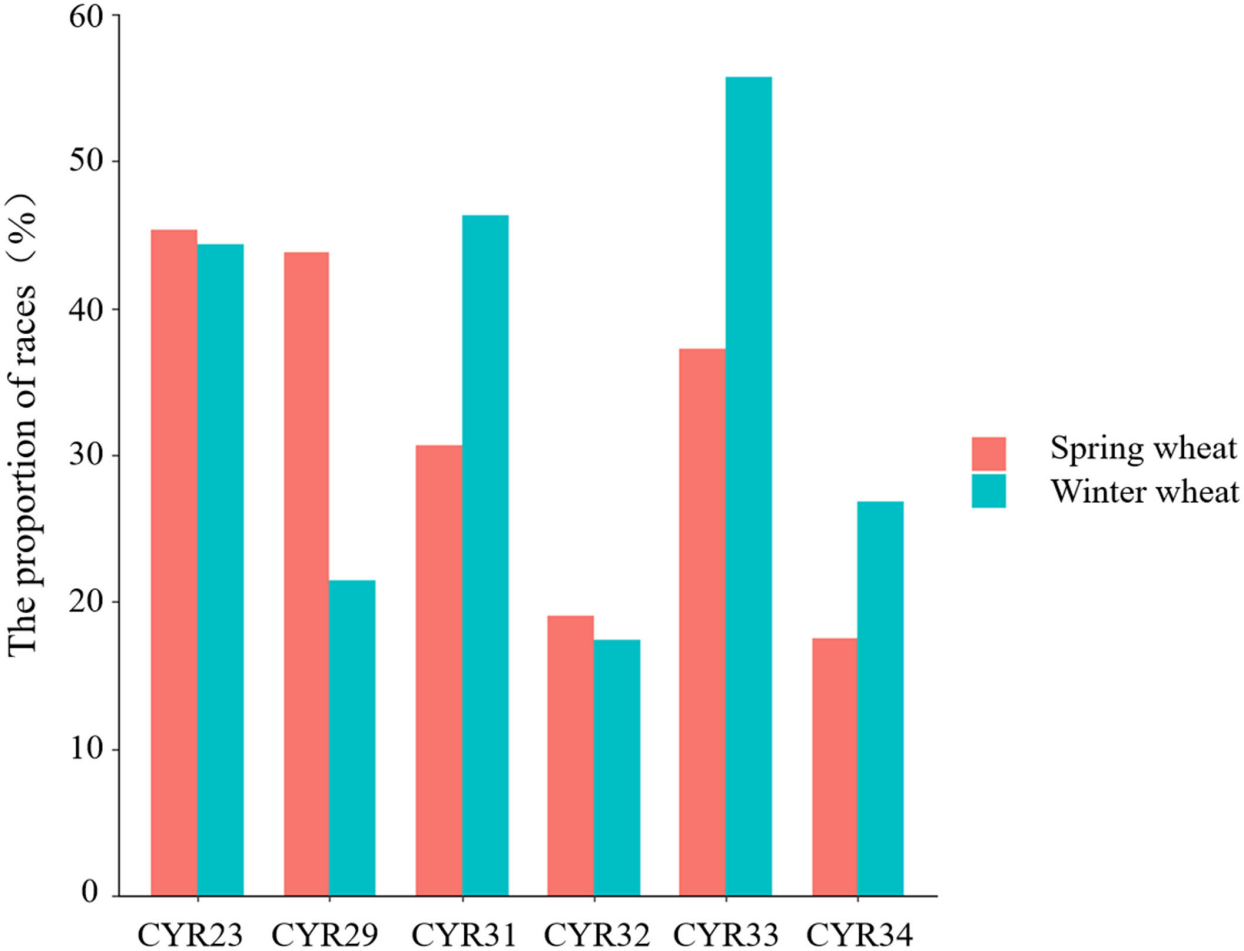
Proportion of spring and winter wheat varieties resistant to six *Pst* races.

Our results indicate that the resistance of spring and winter wheat to CYR23 and CYR32 was similar. While the probability of spring wheat possessing resistance to CYR29 was significantly higher than that of winter wheat (**Figure 2**), the probability of winter wheat possessing resistance to CYR31, CYR33, and CYR34 was significantly higher than that of spring wheat (**Figure 2**).

### 3.3 Molecular detection of resistance genes in spring wheat

**Figure 3:**
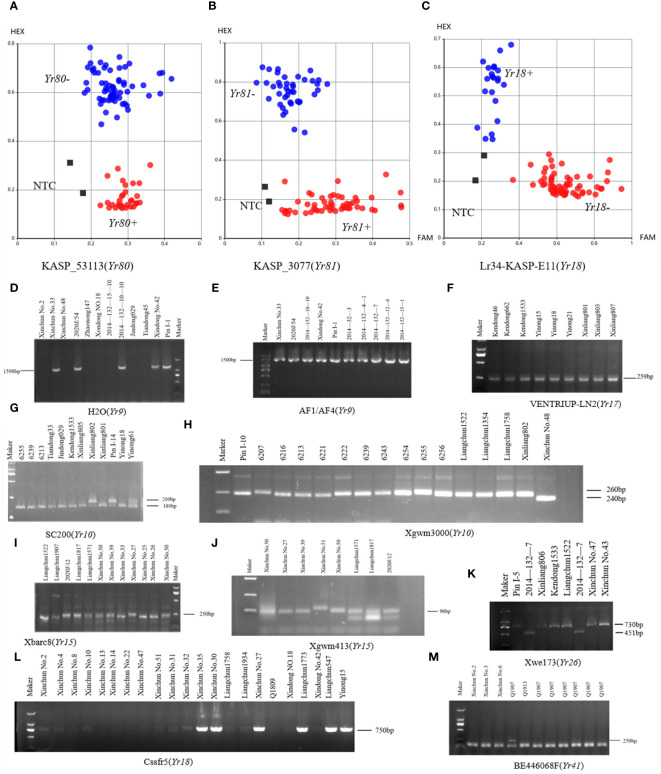
Results of PCR amplification of wheat *Yr* gene. **(A–C)** KASP cluster plots of *Yr80, Yr81*, and *Yr18* genotyping. **(D–M)** Electrophoretograms of the primers for *Yr9*, *Yr10*, *Yr15*, *Yr17*, *Yr18*, *Yr26*, and *Yr41* resistance genes.

Molecular markers closely linked to the *Yr5*, *Yr9*, *Yr10*, *Y15*, *Yr17*, *Yr18*, *Yr26*, *Yr41*, *Yr80*, and *Yr81* genes were used to identify potential *Yr* genes in our spring wheat panel (**Figure 3**; **Supplementary Table S1**). Spring wheat varieties Xinchun No. 33 and 2020J/54 were identified as potential carriers of *Yr9.* The *Yr10*-linked marker amplified the target band in 17 (12.41%) varieties. We identified 21 (15.32%) varieties potentially carrying *Yr15*. The VENTRIUP-LN2 marker, which is closely linked to *Yr17*, amplified the target band in 61 (44.52%) varieties. The KASP marker, which is closely linked to *Yr18*, amplified the target band in 10 (7.30%) varieties (**Figure 3C**). BE446068F, which is closely linked to *Yr41*, was amplified in 10 (7.30%) varieties. The KASP marker, closely linked to *Yr80*, was amplified in 79 (57.66%) varieties (**Figure 3A**), while the KASP marker, closely linked to *Yr81*, was amplified in 32 (23.36%) varieties (**Figure 3B**). *Yr26* and *Yr5* was not detected in all spring wheat varieties. *Yr17* and *Yr80* genes exhibited the highest frequency of distribution in spring wheat.

### 3.4 Molecular detection of resistance genes in winter wheat

In the winter wheat panel, 40 (26.85%) varieties were identified as potential carriers of *Yr9*. The *Yr10* marker amplified the target band in 20 (13.42%) varieties. We identified 7 (4.7%) varieties potentially carrying *Yr15*. The VENTRIUP-LN2 marker amplified the target band in 143 (95.97%) varieties. The KASP marker, which is closely linked to *Yr18*, amplified the target band in 15 (10.03%) varieties (**Figure 3C**). Furthermore, 2014-132-7 was found to harbor the *Yr26* coding region, where the closely linked WE173 marker amplified the target band. BE446068F was amplified in 6 (4.03%) varieties. The KASP marker, closely linked to *Yr80*, was amplified in 38 (25.50%) varieties (**Figure 3A**). Furthermore, 54 (36.24%) varieties amplified the KASP marker, which is closely linked to *Yr81* (**Figure 3B**; **Supplementary Table S2**). Notably, as observed for spring wheat varieties, *Yr17* and *Yr80* also showed the highest frequency of distribution in winter wheat.

### 3.5 Distribution of *Yr* genes in spring and winter wheat

**Figure 4:**
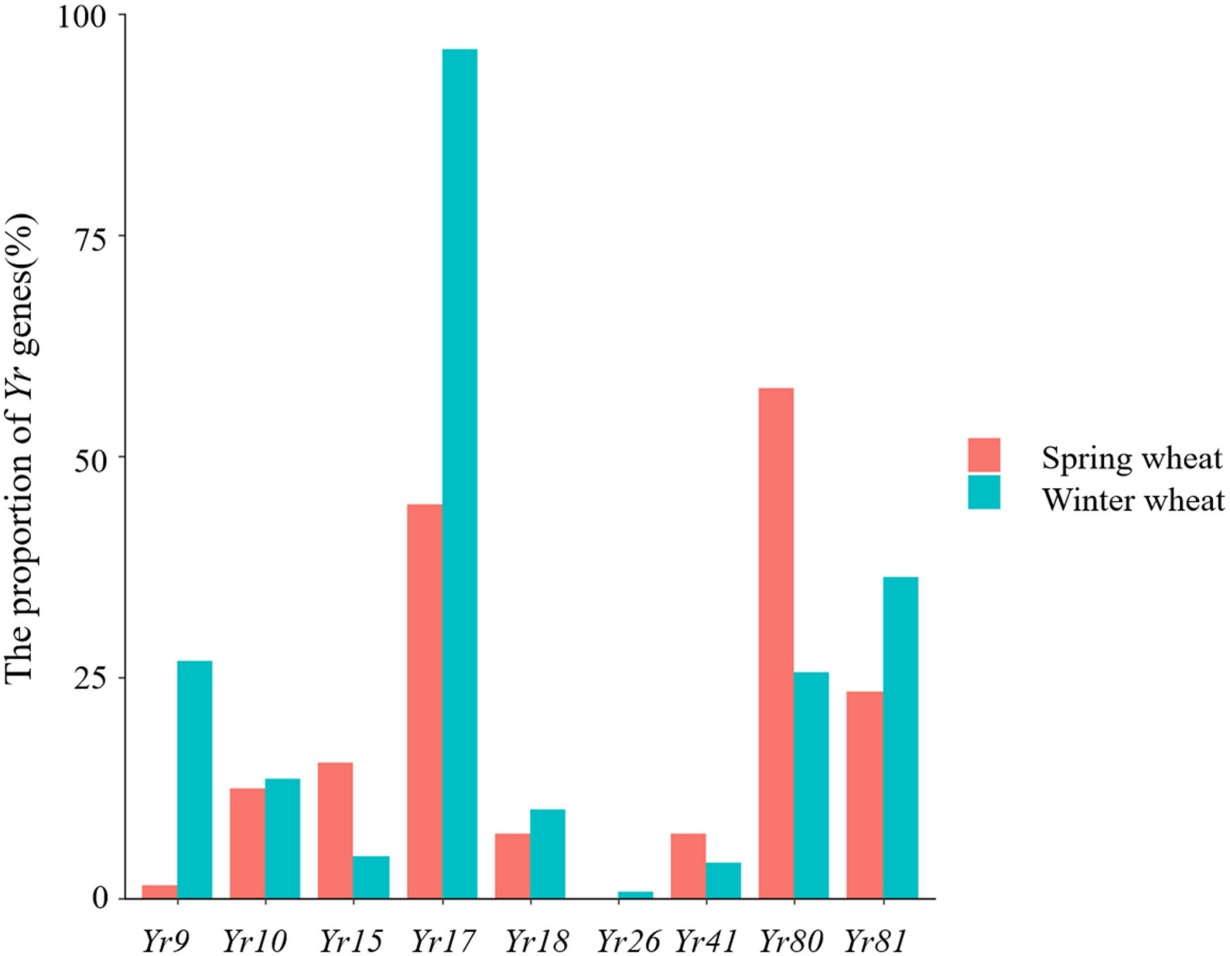
Proportion of *Yr* genes detected in winter and spring wheat varieties.

The results of our *Yr* gene distribution analysis are summarized in **Figure 4**. Specifically, we observed that the proportions of winter wheat expressing *Yr9*, *Yr17*, *Yr18*, and *Yr81* were 26.85%, 95.97%, 10.07%, and 36.24%, respectively. These genes were significantly more prevalent in winter than in spring wheat, whereas the proportion of winter wheat varieties carrying *Yr10* (13.42%) was slightly higher than spring wheat varieties. The proportions of *Yr15* (15.32%) and *Yr41* (7.3%) in spring wheat were slightly higher than in winter wheat, whereas the proportion of *Yr80* (57.66%) in spring wheat was significantly higher than in winter wheat.

### 3.6 Multi-gene combination analysis

**Figure 5:**
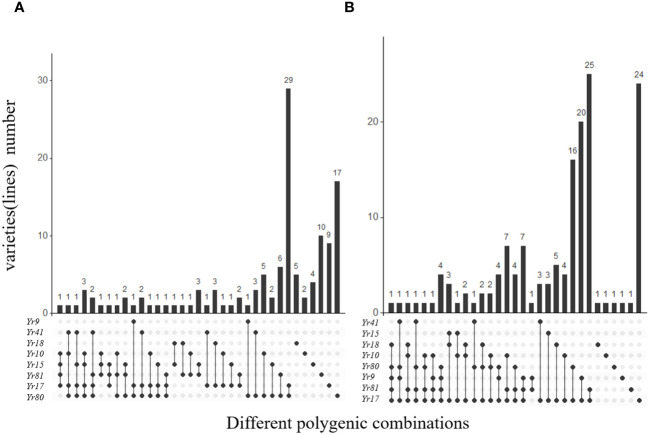
Comparative analysis of *Yr* genes found in 137 spring and 149 winter wheat varieties from Xinjiang. **(A)** Gives the combinations of different *Yr* genes in spring wheat, and **(B)** Gives the combinations of different *Yr* genes in winter wheat.

To further investigate the distribution of resistance genes in different wheat varieties, we assessed the number of varieties containing aggregates of 2, 3, 4, and 5 *Yr* genes in spring and winter wheat (**Figure 5**). The number of multi-gene combined varieties of winter wheat was higher than that of spring wheat. In spring wheat, *Yr17*+*Yr80* was found in 29 of the 137 varieties. However, there were no varieties with 5 or more *Yr* genes (**Figure 5A**). Similarly, *Yr17*+*Yr81* was found in 25 of the 149 winter wheat varieties. Notably, we identified an aggregate in Pin I-9 containing *Yr17*, *Yr81*, *Yr9*, *Yr80*, and *Yr18* (**Figure 5B**).

## 4 Discussion

We assessed disease resistance phenotypes against 6 races of *Pst* in a wheat panel that contained 286 spring and winter wheat varieties found in Xinjiang. Our results show that spring wheat has strong resistance to early popular races CYR23 and CYR29 but weak resistance to currently popular races CYR31, CYR32, CYR33, and CYR34. Only 24 varieties were resistant to CYR34. The resistance of winter wheat to predominant races was generally stronger than that of spring wheat. The number of winter wheat varieties resistant to CYR33 was significantly higher than that of other races, followed by resistance to CYR31 and CYR34, two of the most virulent *Pst* races (Chen et al., 2023; Ma et al., 2023). As the planting area of winter wheat in Xinjiang has expanded, host plants for *Pst* are maintained through summer and winter, allowing the pathogen to complete its annual cycle. This makes it particularly important to select winter wheat varieties with high resistance to stripe rust at the seedling stage, which cannot only reduce stripe rust in autumn plantings of winter wheat, but also prevent propagation of the disease in spring wheat.

To identify potential resistance loci in wheat, it is critical to assess resistance at the seedling stage in a climate chamber, followed by field experiments with artificial inoculations. This conventional method is limited by natural conditions and requires multiple generations to produce resistant cultivars. Molecular marker-assisted selection (MAS) is an effective tool to guide breeding for heritable resistance traits in breeding cultivars (Song et al., 2023). Here, we applied MAS to assess the distribution of wheat resistance genes in varieties from Xinjiang, to better understand endogenous resistance, and to identify potential loci that could be bred into agricultural lines for improved resistance.

The resistance genes *Yr5* and *Yr15* have been reported to convey strong all-stage resistance to the races of stripe rust in China (Zeng et al., 2014). However, *Yr5* was not detected in our panel, and *Yr15* was identified in only a few varieties (Pin I-6, 1-8-2, Liangchun1817). Among wheat varieties that carried *Yr15*, only Liangchun1817 showed resistance to all races at the seedling stage, which may be due to false *Yr15* positives in the susceptible varieties. Based on a previous study, *Yr5* is rarely used in wheat breeding in China, and *Yr15* has only been detected in one study (Zeng et al., 2014). However, Kazakhstan, which is adjacent to Xinjiang, has high frequencies of *Yr5* and *Yr15*, suggesting active breeding efforts towards *Yr5-* and *Yr15*-positive cultivars (Kokhmetova et al., 2021).

The highly virulent race CYR34 has overcome resistance previously conveyed by *Yr26* (Han et al., 2010). Based on a previous study of wheat in Xinjiang, the frequency of *Yr5* is approximately 100% in spring and winter wheat, and that of *Yr26* is 84.15% (Zhang et al., 2023). This is in contrast to our results, where we did not identify *Yr5* in either wheat population, and similarly no *Yr26* gene in spring wheat and only one *Yr26* gene in a winter wheat line (2014-132-7). This may be due to the use of different molecular markers, and possibly false positives or negatives. We did identify one winter wheat variety with an aggregate containing *Yr26*, *Yr9*, and *Yr17* that displayed strong resistance to CYR34, indicating that this polygene combination can supplement *Yr26-*mediated resistance against CYR34. In Indian wheat, *Yr26* and *Yr10* have high frequencies in the distribution of resistance genes (Rani et al., 2019). While resistance gene distributions in wheat from Xinjiang were similar to those from the rest of China, there were notable differences between Xinjiang and neighboring India and Kazakhstan. This is expected, given the completely different cultivated varieties based on climate and breeder preference. We suggest that a comparative analysis of wheat *Yr* genes and phenotypes in different regions be performed to reveal the breeding trajectories of different regions.

*Yr18* is located on chromosome 7D and codes for an APR gene that slows *Pst* infections in the adult stage. Additionally, *Yr18* conveys resistance to leaf rust, stem rust, and powdery mildew (Singh et al., 2000). While the frequency of *Yr18* detection in Chinese wheat landraces is generally high, *Yr18* is largely absent from Xinjiang wheat. This could be attributed to the infrequent use of wheat landraces in Xinjiang wheat breeding. Therefore, combination of *Yr18* with other ASR and APR genes may significantly improve the stripe rust resistance of Xinjiang wheat varieties.

*Yr5*, *Yr10*, *Yr15*, *Yr26*, and *Yr18* genes exhibit partial resistance to stripe rust and occur infrequently in Xinjiang wheat varieties. *Yr26* and *Yr10* genes have been overcome by CYR34, yet recent research indicates that the predominant stripe rust population in Xinjiang belongs to the Su 11 pathogenic type (Chen et al., 2023; Ma et al., 2023). *Yr26* and *Yr10* retain their efficacy in controlling stripe rust in Xinjiang. Wheat varieties in Xinjiang carrying *Yr10* and *Yr26*, such as 2014-129-1-6, Kendong47, and 2014-132-7, can effectively manage the prevalent stripe rust type in the region. Given their strong resistance to Xinjiang stripe rust, these genes can be incorporated into future breeding programs for Xinjiang wheat varieties.

With the source variation of stripe rust and the emergence of new virulence factors, many single resistance genes have been overcome. In a previous analysis of wheat resistance genes, polymerization or stacking of multiple genes has been shown to effectively improve resistance (Zhang et al., 2023). In this study, we found that most of the wheat varieties in Xinjiang contained two or more *Yr* genes. We found high prevalence of *Yr80*, *Yr81* and all-stage resistant *Yr17* in both spring and winter wheat, mainly as components of polygene doublets of *Yr17*+*Yr80* and *Yr17*+*Yr81*, as well as some other combinations. The results of wheat resistance to stripe rust in Sichuan, the Huang-Huai-Hai region, and Yunnan were similar to ours, indicating that polygene polymerization, specifically *Yr17*+*Yr81*, can improve the resistance to wheat stripe rust (Li et al., 2016; Wang et al., 2018; Guan et al., 2020; Huang et al., 2020; Xi et al., 2021). *Yr17* from the 2NS/2AL translocation line of *Aegilops* has all-stage resistance and the distribution frequency in spring and winter wheat, however, all the prevalent *Pst* races have overcome *Yr17*-mediated resistance. Since the emergence of CYR29, only wheat varieties carrying *Yr9* alone have lost their resistance. In this study, we found that wheat varieties with *Yr9*+*Yr17* showed strong resistance to wheat stripe rust races. Notably, we identified two *Yr9*+*Yr17* winter wheat lines, 2014-132-4-5 and 2014-129-13-9, that displayed seedling resistance to all 6 races, indicating that the combination of *Yr9* and *Yr17* can effectively defend against all prevalent *Pst* races.

## 5 Conclusions

Our comprehensive analysis of 286 varieties of spring and winter wheat in Xinjiang provides valuable insights into the dynamics of stripe rust resistance. Spring wheat demonstrates robust resistance to early races CYR23 and CYR29 but shows weak resistance against contemporary races CYR31, CYR32, CYR33, and CYR34, with only 24 varieties displaying resistance to CYR34. Conversely, winter wheat exhibits stronger resistance, particularly against CYR33, CYR31, and CYR34, highlighting the importance of selecting resistant winter wheat varieties to curtail stripe rust propagation into spring wheat.

Utilizing MAS, we identify the distribution of key resistance genes, revealing variations in the prevalence of genes such as *Yr5*, *Yr15*, *Yr26*, and *Yr18* compared to previous studies. The absence of *Yr5* and limited detection of *Yr15* suggest regional disparities in breeding priorities, as seen in neighboring Kazakhstan. The emergence of highly virulent CYR34, overcoming *Yr26*-mediated resistance, underscores the evolving nature of stripe rust and the need for adaptable resistance strategies.

Our findings emphasize the significance of polygene combinations in enhancing resistance. Notably, the combination of *Yr17* and *Yr81* demonstrates effectiveness in resisting stripe rust, as observed in the prevalence of *Yr17*+*Yr80* and *Yr17*+*Yr81* combinations. Furthermore, the synergy between *Yr9* and *Yr17* proves highly effective against all prevalent *Pst* races, offering promising avenues for breeding resilient wheat varieties.

The absence of *Yr18* in Xinjiang wheat, despite its widespread presence in Chinese landraces, underscores the potential for improved resistance through strategic gene combinations. We propose that combining *Yr18* with other APR genes could significantly enhance stripe rust resistance in Xinjiang wheat. In conclusion, our study contributes crucial data for breeding durable and resilient wheat varieties in Xinjiang, offering insights into regional resistance dynamics and highlighting the efficacy of polygene combinations in mitigating the impact of evolving stripe rust races. These findings can guide future breeding strategies and foster international collaboration for a more comprehensive understanding of wheat resistance across diverse regions.

## Data availability statement

The raw data supporting the conclusions of this article will be made available by the authors, without undue reservation.

## Author contributions

HL: Writing – original draft, Conceptualization, Data curation, Formal analysis. YS: Conceptualization, Writing – original draft. HY: Formal analysis, Writing – original draft. DF: Data curation, Formal analysis, Writing – original draft. CR: Conceptualization, Data curation, Writing – original draft. FD: Data curation, Methodology, Writing – review & editing. YiL: Formal analysis, Funding acquisition, Writing – review & editing. NS: Project administration, Validation, Writing – review & editing. LC: Investigation, Methodology, Writing – review & editing. GL: Funding acquisition, Resources, Writing – review & editing. HW: Funding acquisition, Writing – review & editing. HG: Funding acquisition, Writing – review & editing. YuL: Methodology, Project administration, Visualization, Writing – review & editing.
